# Effects of Lanthanum on the Photosystem II Energy Fluxes and Antioxidant System of *Chlorella Vulgaris* and *Phaeodactylum Tricornutum*

**DOI:** 10.3390/ijerph16122242

**Published:** 2019-06-25

**Authors:** Dong Sun, Ning He, Qi Chen, Shunshan Duan

**Affiliations:** 1 Research Center of Hydrobiology, Key Laboratory of Aquatic Eutrophication and Control of Harmful Algal Blooms of Guangdong Higher Education Institute, Jinan University, Guangzhou 510632, China; jnu_sundong@163.com (D.S.); cq92088@outlook.com (Q.C.); 2 Colleges of Life Science and Resource and Environment, Yichun University, Yichun 336000, China; hening2010@163.com

**Keywords:** Antioxidant system, bait algae, photosystem II, pollution, rare earth elements

## Abstract

The rare earth elements are widely used in agricultural and light industry development. They promote the growth of crop seedlings, enhance root development and change the metal properties. Due to the large amount of rare earth minerals mined in China, rare earth elements have been detected in both coastal and estuary areas. They cause pollution and threaten the health of aquatic organisms and human beings. This study investigates the effects of lanthanum on two marine bait algae, and analyzes the changes in the photosynthetic and antioxidant systems of the two algae. The results show that rare earth elements have significant inhibitory effects upon the two algae. The OJIP kinetic curve value decreases with an increasing concentration of La(NO_3_)_3_ ·6H_2_O. The parameters of the fluorescence value were analyzed. The ABS/RC increases and the DI_0_/RC decreases during the first 24 h after exposure. The effects on the photosynthetic and antioxidant systems at low concentrations (both EC_10_ and EC_20_) show that the TR_0_/ABS increases, and the ET_0_/RC, ABS/RC, and DI_0_/RC has a decreasing trend after 30 min. However, after 24 h, normal levels were restored. In addition, the study finds that the TR_0_/ABS increases after 24 h, leading to an increase in reactive oxygen species. The antioxidant system analysis also confirms the increase in the activities of antioxidant enzymes, such as SOD and GSH. The experiment is expected to support the marine pollution of rare earths and the theoretical data of the impact on marine primary producers.

## 1. Introduction

The rare earth elements (REEs) are a series of metal elements with similar physical and chemical properties. China holds a near monopoly on the production of REEs in many years, and contains the largest mineral deposits in the world [1,2]. In the last several decades, the exploitation and use of rare earth sources improves economic and agricultural development. REEs have received increasing attention in recent years because they contaminate the environment and cause potential harm to organisms [3,4,5]. Some studies report that REEs are largely found in the air [6], soil [7], sediments [8], and water [9]. Moreover, they are also detected in vegetables [10], fishes [11], and especially in human urine and hair [12,13]. Some REEs cause damage to organisms and also cause human diseases. Other studies reported that REEs accumulate in organisms [14] and are toxic at environmental concentrations. Hence, the adverse effects of REEs on aquatic organisms need further exploration. 

Algae are an important part of oceans and the primary producer in the marine ecosystem. Hence, the growth of algae is often used to assess the pollution conditions in the water environment. REEs inevitably affect the algae in the water. Some reports showed that a high concentration of REEs inhibits the growth of plants and algae [15,16]. A few studies have been conducted on the toxic effects of REEs on algae. However, whether photosystem (PS) II was damaged or the antioxidant system was disordered is still unclear [17,18]. Therefore, the inhibitory effects of REE exposure need to be investigated further. 

The photosynthetic processes support the biochemical reactions needed for the growth of algae. Thus, changes in external environmental conditions disrupt the balance for PS and the ability to use light energy [19]. Some reports show that the heavy metals damage plant chloroplast or photosynthetic organs and reduce photosynthetic efficiency [20]. The OJIP test is used to assess the response of plants and algae to environmental stresses and the effect on PS II [21,22]. However, a few studies are performed on the effects of REEs on the algal chlorophyll *a* fluorescence, especially the photochemical reaction. When the algal cells are subjected to pollution stress, the oxygen in the cells is converted into the damaging reactive oxygen species (ROS). The ROS and their products have a strong oxidizing ability and destroy many biomolecules [23,24]. The superoxide dismutase (SOD), glutathione (GSH), peroxidase (POD), and catalase (CAT) belong to the antioxidant enzyme system and indicate the extent of algal cell damage [25]. Therefore, many studies have used these indicators to reflect the algae conditions [26,27,28]. 

The investigations on the toxic effects of rare earth pollutants on the photosynthetic and antioxidant responses of algae are limited. Thus, this study aimed to: (1) Investigate the toxic effect of La(NO_3_)_3_·6H_2_O on *Chlorella vulgaris* and *Phaeodactylum tricornutum*, including the growth, photosynthetic, and antioxidant responses; and (2) to analyze whether the inhibition effect was due to the destruction of the photosynthetic system or of the oxidation system. 

## 2. Materials and Methods

### 2.1. Algae Culture 

Marine microalgae *C. vulgaris* and *P. tricornutum* were obtained from the Research Center of Hydrobiology, Jinan University, Guangzhou, China. These two marine microalgae cultures were obtained using artificial seawater enriched with a modification of f/2 medium without ethylenediaminetetraacetic acid (EDTA), (the initial pH was adjusted to 8.0 with 0.5 mol·L^−1^ HCl or NaOH). EDTA greatly decreases the toxicity of elements due to the chelating properties of the molecules [29]. The f/2 media contained all of the essential elements and trace elements necessary for the algae growth. Culture of *C. vulgaris* and *P. tricornutum* was carried out in 2-L flasks, with each flask containing 1 L of the medium. The culture was illuminated with fluorescent light at a light intensity of 35 μmol·s^−1^·m^−2^ at the surface of the culture medium, while with a 12/12-h light/dark cycle in an environmental chamber at 23 °C ± 1 °C. This culture period had 3–5 cycles (7–9 days per cycle) to activate the microalgae to follow on the experiment. 

### 2.2. Experimental Design 

*C. vulgaris* and *P. tricornutum* were grown in the f/2 medium with La(NO_3_)_3_ ·6H_2_O. The effects of La(NO_3_)_3_·6H_2_O at concentrations of 0, 2, 4, 6, 8, and 10 mg·L^−1^ on microalgae growth were assessed and fluorescence transient measurements were conducted. Cultivations were performed in 150-mL flasks (100-mL culture medium) in triplicate at 23 °C ± 1 °C and 35 μmol·s^−1^·m^−2^ under a 12/12-h light/dark cycle. The inoculation density was 2 × 10^5^ cell·mL^−1^. The flasks were shaken by hand and their places were randomly changed three times a day. The algae cell density was determined with a hemocytometer (Qiujing Company, Shanghai, China) three times for each sample under a 400× magnification of the microscope (BX53, OLYMPUS, Japan). 

*C. vulgaris* and *P. tricornutum* were exposed to La(NO_3_)_3_·6H_2_O for 96 h. During this period, the growth and fluorescence transient measurements of the two algae were determined daily and used to investigate the inhibitory effect of La(NO_3_)_3_·6H_2_O on algae. After 96 h, the EC10 and EC20 of *C. vulgaris* and *P. tricornutum* were calculated according to the dose–effect formula. 

The extent of damage caused by EC10 and EC20 of La(NO_3_)_3_·6H_2_O to the photosynthetic system II and the antioxidant system of the two algae was observed. Meanwhile, the emergency response and strategy of algae against metal pollutants were examined.

### 2.3. Measurements of Fluorescence Transient

The fluorescence transient was measured using a Handy Plant Efficiency Analyzer (Hansatech Ltd., UK). Measurements were performed on 2-mL samples in 6-mm-diameter transparent vials, which were dark-adapted for 15 min at room temperature. The fluorescence transients were recorded for up to 2 s in a logarithmic time scale. The selected OJIP test parameters (Table 1) to quantify the fluorescence were calculated from the original data using the formula described by previous research [21,22,30].

The following equations were used to evaluate the energy fluxes in PS II:

The trapping probability or the maximum efficiency of PS II photochemistry, TR_0_/ABS, or *F*_v_/*F*_m_ = (*F*_m_ − *F*_50μs_)/*F*_m_

The effective antenna size of an active RC or absorption flux per RC, ABS/RC = (*M*_0_/*V*_j_)/(TR_0_/ABS), where *V*_j_ = (*F*_2 ms_ − *F*_50 μs_) /(*F*_m_ − *F*_50 μs_)

The effective dissipation of an active RC, DI_0_/RC = (ABS/RC) − (*M*_0_/*V*_j_)

The electron transport rate in an active RC, ET_0_/RC = (*M*_0_/*V*_j_) × (1 − *V*_j_)

### 2.4. Extraction and Determination of Protein and Antioxidase Levels

After exposure to EC_10_ and EC_20_ for 96 h, the flasks with microalgae were taken out and shaken by hand. Then, 40 mL of microalgae cells in each flask were sampled after 1 h (the dead cells precipitated to the bottom) and centrifuged at 7500 rpm for 10 min at 4 °C. The liquid supernatant was discarded, followed by ultrasonic (UH-950B, Tianjing, China) disruption (5-s ultrasound and 5-s stop) after adding 9 mL of saline for 30 min in the ice-water bath. These samples were centrifuged at 7500 rpm for 10 min at 4 °C, and the liquid supernatant was preserved in vials in the ice-water bath for further experiments.

The total protein concentration was determined with an assay kit purchased from the Nanjing Jiancheng Bioengineering Institute according to bicinchoninic acid assay [31]. 

The peroxidase (POD) activity was determined with an assay kit purchased from Nanjing Jiancheng Bioengineering Institute following the methods described by Montavon and Bortlik [32]. The reaction was initiated by adding the extract, and the absorbance was read at the 420 nm wavelength using a UV–visible spectrophotometer (UV-2450, Shimadzu, Japan) for 30 min at 37 °C. One unit of POD activity was defined as 1 μg H_2_O_2_·mg^−1^ protein·min^−1^.

The activity of superoxide dismutase (SOD) was determined using an assay kit of WST-1 purchased from Nanjing Jiancheng Bioengineering Institute. Water-soluble tetrazolium was used as the detector of the superoxide radicals generated by xanthine oxidase and hypoxanthine in the presence of a range of concentrations of SOD [33]. One unit of SOD activity was defined as the amount of enzyme inhibiting 50% of WST-1 photoreduction [34]. 

The catalase (CAT) activity was determined using an assay kit purchased from the Nanjing Jiancheng Bioengineering Institute. The absorbance at 405 nm of the reaction liquid was measured with a UV–visible spectrophotometer as described by Góth [35]. One unit of CAT activity was defined as 1 μmol H_2_O_2_·mg^−1^ protein·s^−1^.

The glutathione (GSH) concentration was determined with an assay kit purchased from the same Nanjing Jiancheng Bioengineering Institute based on the reaction of 5.5’-dithio-bis (2-nitrobenzoic acid) with sulfhydryl compound as described by Anderson [36]. The content of GSH was calculated based on the standard curve.

### 2.5. Data Analysis and Statistics

Data were expressed as means and standard deviations replicated three times. Origin 8.0 (Origin Lab, Northampton, MA) was used to construct the figures. The data were analyzed using the one-way analysis of variance (ANOVA). If the statistical test was significant at *p* < 0.05, the differences were compared using the LSD comparison method with the SPSS 17.0 for Windows (SPSS, Chicago, IL, USA).

## 3. Results 

### 3.1. The Toxicity Effect of La(NO_3_)_3_·6H_2_O on the Two Algae

**Figure 1:**
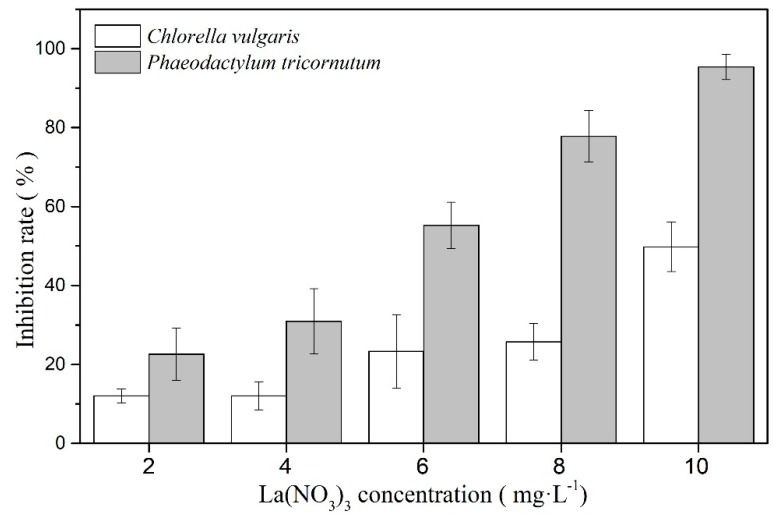
Inhibition rate for various concentrations of La(NO_3_)_3_·6H_2_O in 96 h.

Five different concentrations of La(NO_3_)_3_·6H_2_O are used to inhibit the growth of the two marine microalgae for 96 h (Figure 1). The results show that La(NO_3_)_3_·6H_2_O has inhibitory effects on both marine microalgae. When the concentrations of La(NO_3_)_3_·6H_2_O increase, the inhibition rates increase. According to the dose–effect formula, the EC_50_ value of *C. vulgaris* and *P. tricornutum* is 10.077 mg·L^−1^ and 5.665 mg·L^−1^, respectively. After 96 h, 10 mg·L^−1^ La(NO_3_)_3_·6H_2_O could inhibit 96% of *P. tricornutum*. Thus, La(NO_3_)_3_·6H_2_O has a strong toxic effect on the two microalgae. 

### 3.2. Effect of La(NO_3_)_3_·6H_2_O on PS II of the Two Microalgae

**Figure 2:**
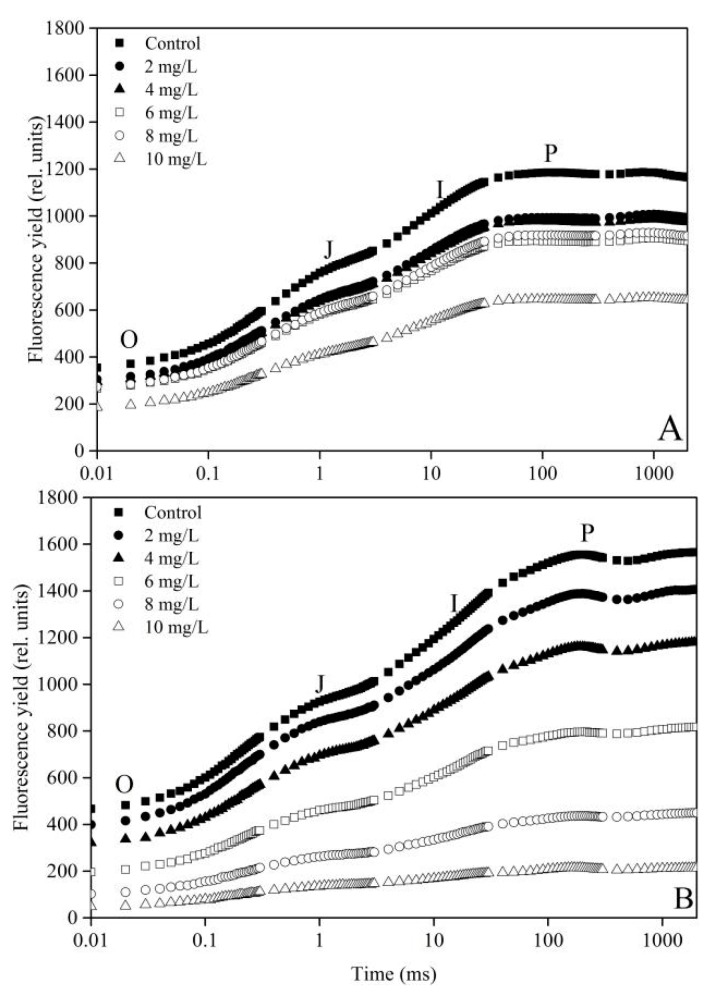
Rapid increase in fluorescence transients for *C. vulgaris* (**A**) and *P. tricornutum* (**B**) exposed to La(NO_3_)_3_·6H_2_O for 96 h.

Figure 2 (OJIP curves) shows the chlorophyll fluorescence transients of *C. vulgaris* and *P. tricornutum* exposed to different concentrations of La(NO_3_)_3_·6H_2_O for 96 h. When the concentration of La(NO_3_)_3_·6H_2_O increases, the fluorescence transients of *C. vulgaris* and *P. tricornutum* significantly decrease, and are lower compared with the control. When the concentration is at 10 mg·L^−1^, maximum fluorescence is observed (*F*_m_ or P point). However, the fluorescence transients of *P. tricornutum* decrease rapidly compared with those of *C. vulgaris* at the same concentration of La(NO_3_)_3_·6H_2_O. These results indicate that *C. vulgaris* is more resistant than *P. tricornutum*.

**Figure 3:**
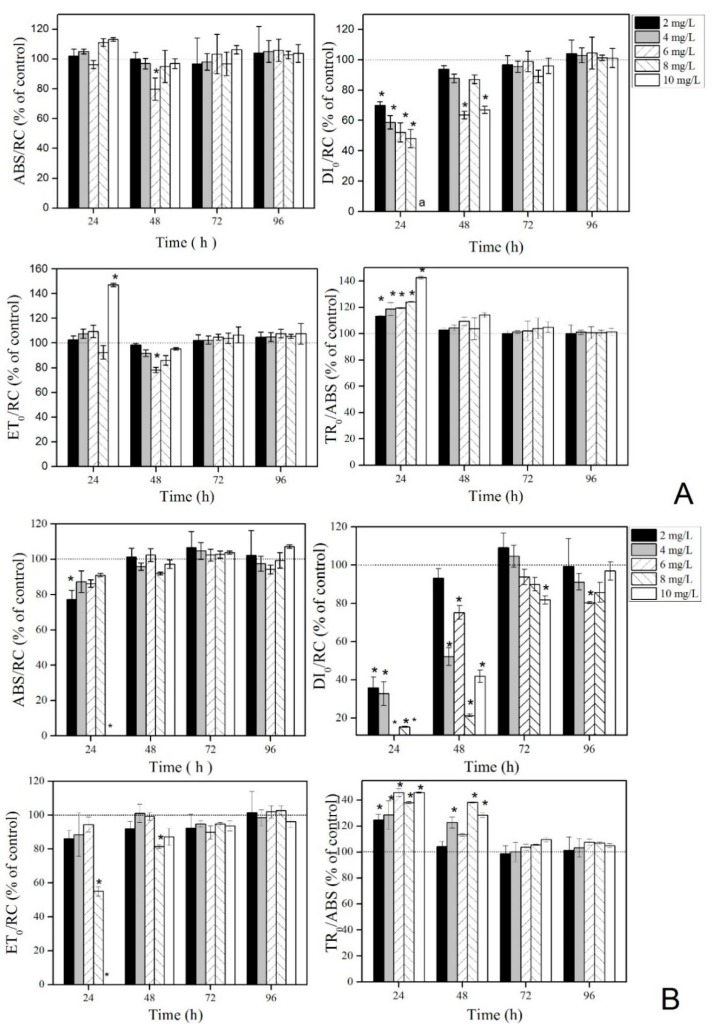
OJIP-test parameters expressed as a percentage of the control when *C. vulgaris* (**A**) and *P. tricornutum* (**B**) were treated with La(NO_3_)_3_·6H_2_O and dark-adapted for 15 min. * Significant difference compared with the control (*p* < 0.05). ^a^ The value was too low to be measured.

As shown in Figure 3A, ABS/RC has no significant difference between groups. DI_0_/RC decreases significantly with an increasing concentration of La(NO_3_)_3_·6H_2_O after 24 h and recovers after 48 h, but the effects at 6 and 10 mg·L^−1^ are still significantly lower compared with the control. After 72 h, no significant difference is found between the groups. ET_0_/RC is significantly higher in the 10 mg L^−1^ group compared with the control after 24 h; the value in the 6 mg·L^−1^ group is lower compared with the control after 48 h. After 72 h, the ET_0_/RC values have no significant difference between groups. TR_0_/ABS increased significantly with increasing concentrations of La(NO_3_)_3_·6H_2_O after 24 h. After 48 h, no significant difference is found between the groups.

As shown in Figure 3B, ABS/RC is lower compared with the control after 24 h. No significant difference is found between groups after 48 h. DI_0_/RC is significantly lower compared with the control and decreases with the decreasing concentration of La(NO_3_)_3_·6H_2_O after 24 h. After 48 h, the DI_0_/RC recovers but is still significantly lower compared with the control. After 72 h, DI_0_/RC returns to the control levels, but is still not stable. ET_0_/RC significantly decreases at a high concentration (10 mg·L^−1^) after 24 h and is significantly lower compared with the control in the 6 mg·L^−1^ group after 48 h. After 72 h, the groups have no significant difference compared with the control. TR_0_/ABS is significantly higher compared with the control before 48 h, and has no significant difference between groups after 72 h. 

### 3.3. Effect of Low Concentrations (EC_10_ and EC_20_) of La(NO3)3·6H_2_O on PS Ⅱ and Antioxidant Enzyme System

**Figure 4:**
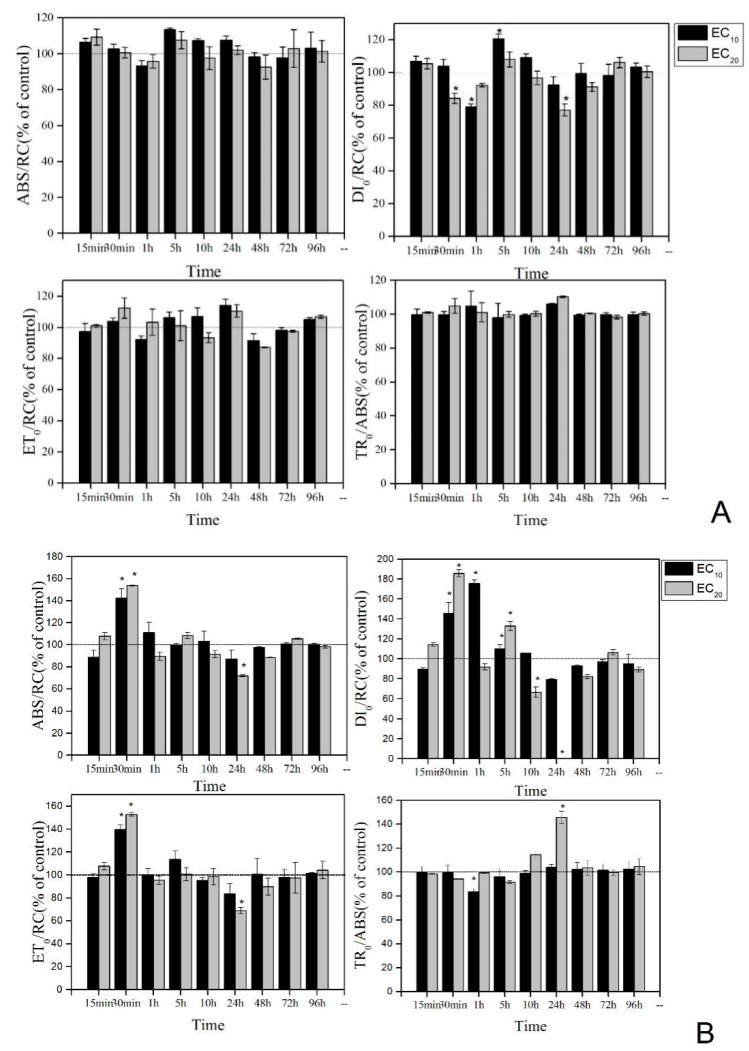
OJIP test parameters expressed as a percentage of the control when *C. vulgaris* (**A**) and *P. tricornutum* (**B**) were treated with EC_10_ and EC_20_ of La(NO_3_)_3_·6H_2_O and dark-adapted for 15 min. *Significant difference compared with the control (*p* < 0.05).

The results indicate that La(NO_3_)_3_·6H_2_O indeed inhibits the growth of algae, but whether the inhibition of algae growth is due to the destruction of photosynthetic or oxidation–reduction system is not clear. Hence, EC_10_ and EC_20_ of La(NO_3_)_3_·6H_2_O are used as the experimental concentration for the next experiment (Figure 4). According to the dose–effect formula calculation, the EC_10_ and EC_20_ of *C. vulgaris* are 3.271 and 6.404 mg·L^−1^, and the EC_10_ and EC_20_ of *P. tricornutum* are 1.262 and 2.282 mg·L^−1^, respectively. 

After adding La(NO_3_)_3_·6H_2_O, the PS II indicators of *C. vulgaris* have wave-shaped disturbances with time. Although ABS/RC fluctuates, no significant difference is found compared with the control. DI_0_/RC significantly decreases after 30 min and 24 h, and significantly increases after 5 h. ET_0_/RC and TR_0_/ABS have no significant difference compared with the control at each time point. 

The effect of La(NO_3_)_3_·6H_2_O is greater on PS II indicators of *P. tricornutum* than on *C. vulgaris*. ABS/RC significantly increases after 30 min and then gradually stabilizes to the control. DI_0_/RC significantly increases from 30 min to 5 h and decreases after 10 and 24 h. After 48 h, DI_0_/RC reaches the level of control. ET_0_/RC significantly increases after 20 min but significantly decreases after 24 h, and then gradually stabilizes to the control. TR_0_/ABS significantly decreases after 1 h for EC_10_ and increases after 24 h for EC_20_. 

**Figure 5:**
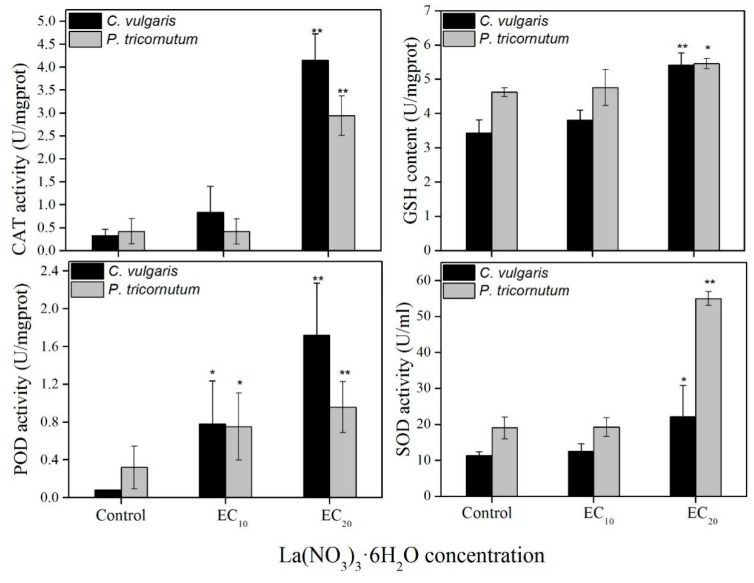
The effect of La(NO_3_)_3_·6H_2_O on antioxidant enzyme (catalase (CAT), peroxidase (POD) and superoxide dismutase (SOD)) activities and glutathione (GSH) content of *C. vulgaris* and *P. tricornutum* after 96 h. * Significant difference compared with the control (*p* < 0.05). ** Significant difference compared with the control (*p* < 0.01).

No significant difference is found in CAT activity, SOD activity, and GSH content at EC_10_; however, the POD activity is higher compared with the control (Figure 5). At EC_20_, the CAT activity, GSH content, POD activity, and SOD activity are significantly higher compared with those of the control, indicating that algae become stressed at EC_20_ of La(NO_3_)_3_·6H_2_O.

## 4. Discussion

In the present study, different concentrations of La(NO_3_)_3_·6H_2_O show a significant inhibitory effect on the two algae. The inhibition rates increase with increasing concentrations of La(NO_3_)_3_·6H_2_O. Tai also shows that all single lanthanides have toxic effects on *Skeletonema costatum* [29]. Also, Goecke reports similar results that 72-h EC_50_ for four REE salts (Ce, Gd, La, and Pr) are between 1.2 and 1.4 mg·L^−1^, and presumes that this inhibitory effect is caused by the nutrient sequestration from the algal growth medium [37]. 

However, Tai reports that the nitrates of REEs have no effects on algae at low concentrations (5–60 μmol·L^−1^) [29]. This finding is inconsistent with the present study in which La(NO_3_)_3_·6H_2_O has an inhibitory effect on *P. tricornutum*, and the EC_10_ and EC_20_ are 1.262 mg·L^−1^ (2.91 μmol·L^−1^) and 2.282 mg·L^−1^ (5.27 μmol·L^−1^), respectively. The results indicate that low concentrations of nitrates of REEs also affect algae. Although the content of La is not high in the environmental water, it can accumulate to a damaging concentration, thereby potentially inhibiting the growth of algae. 

When the growth of algae is inhibited by stress, it indicates that the photosynthetic system of algae has been damaged [20]. The OJIP curve values (O, OJ, JI, and IP) decrease with the increasing concentrations of La(NO_3_)_3_·6H_2_O in the present study. The O point reduction indicates that the number of algae cells decreases. The decrease in JI and IP indicates that the high concentrations of La(NO_3_)_3_·6H_2_O inhibit the photoactivation of PS II. This might be due to the increase in non-Q_B_ in the PS II, blocking the transfer from Q_A_ to Q_B_ [38]. This is consistent with the finding that the photosynthetic system of algae is changed by polycyclic aromatic hydrocarbon stress [39]. However, the photosynthetic parameters are analyzed in the first 24 h in this study. DI_0_/RC decreases with the increasing concentration of La(NO_3_)_3_·6H_2_O, while TR_0_/ABS increases with the increasing concentration of La(NO_3_)_3_·6H_2_O. It showed that the trapping dose of PS II increases on La(NO_3_)_3_·6H_2_O exposure, but the dissipated dose reduces. This result is inconsistent with the exposure of higher plants to aluminum (Al) [21]. This might be because REEs promote the growth of plants and algae. 

To understand the changes in the fluorescence parameter values of PS II, the effects of low concentrations of La(NO_3_)_3_·6H_2_O on the two algae are continuously monitored. The results show that, after 30 min, TR_0_/ABS increases, and ET_0_/RC, ABS/RC, and DI_0_/RC decrease on exposure to La(NO_3_)_3_·6H_2_O. This indicates that the trapping dose decreases, while the total excitation, dissipation dose, and electron transport rate increases on La(NO_3_)_3_·6H_2_O exposure. It also reveals the decreased activity of electron transfer chains in photosynthetic systems after algae are stressed by contamination. This might be caused by the trapping and blocking of electron transfer of Q_A_ [22] and also an inactivation of the PS II active center [40]. This result is consistent with the effect of low concentrations of nonylphenol on the *Chlamydomonas reinhardtii* strain CC125 and on *Microcystis aeruginosa* CPCC632 [41]. However, after 24 h, the trapping values (TR_0_/ABS) reach a peak, and excessive absorption leads to the production of ROS, which causes peroxidation damage to the photosynthetic apparatus [42]. Therefore, after 96 h, the antioxidant enzyme values at EC_20_ are significantly higher compared with the control, indicating that the antioxidant defense system was activated after the algae was stressed by La(NO_3_)_3_·6H_2_O. Other studies also show that the SOD activity and GSH content increase in the two algae to reduce the harm caused by active oxygen [43,44].

## 5. Conclusions 

In conclusion, La(NO_3_)_3_·6H_2_O had a significant inhibitory effect on the two algae. In the first 48 h, the inhibition by La(NO_3_)_3_·6H_2_O is mainly caused by the destruction of PS II and the antioxidant system. After 48 h, the PS II restores to the control levels. However, the oxidative stress remains on exposure to La(NO_3_)_3_·6H_2_O, and affects the growth of algae. 

## Figures and Tables

**Table 1:** Parameters used in the analysis of OJIP fluorescence induction dynamics curves [21,22,30].

Terms	Illustration
*F_0_*	Minimal recorded fluorescence intensity
*F_m_*	Maximal recorded fluorescence intensity
*F*_50 μs,_ *F*_2 ms_	Fluorescence intensities at 50 μs and 2 ms
OJIP	O phase (*F*_20 μs_*/F_0_*), J phase (*F*_2 ms_), I phase (*F*_30 ms_) and P phase (*F_m_*)
*V_j_*	Relative variable fluorescence intensity at the J-step
*M_0_*	Approximated initial slope of the fluorescence transient
RC	Reaction center of photosystem II
